# A Promising Copper(II) Complex as Antifungal and Antibiofilm Drug against Yeast Infection

**DOI:** 10.3390/molecules23081856

**Published:** 2018-07-26

**Authors:** Fabiana Gomes da Silva Dantas, Adriana Araújo de Almeida-Apolonio, Renata Pires de Araújo, Lis Regiane Vizolli Favarin, Pamella Fukuda de Castilho, Fernanda de Oliveira Galvão, Terezinha Inez Estivalet Svidzinski, Gleison Antônio Casagrande, Kelly Mari Pires de Oliveira

**Affiliations:** 1 Faculty of Health Sciences, Federal University of Grande Dourados, Dourados, MS 79804-970, Brazil; fabianasilva@ufgd.edu.br (F.G.d.S.D.); pamellafcastilho@gmail.com (P.F.d.C.); feergalvao@hotmail.com (F.d.O.G.); 2 Faculty of Medicine, Federal University of Mato Grosso do Sul, Campo Grande, MS 79070-900, Brazil; aaraujo.a@hotmail.com; 3 Faculty of Exact Sciences and Technology, Federal University of Grande Dourados, Dourados, MS 79804-970, Brazil; renataaraujo@ufgd.edu.br; 4 Institute of Chemistry, Federal University of Mato Grosso do Sul, Campo Grande, MS 79074-460, Brazil; lisregiane@hotmail.com; 5 Department of Clinical Analysis and Biomedicine, State University of Maringá, Maringá, PR 87020-900, Brazil; terezinha.svidzinski@gmail.com; 6 Faculty of Biological and Environmental Science, Federal University of Grande Dourados, Dourados, MS 79804-970, Brazil

**Keywords:** antifungal, ames test, biofilm, copper(II), 2-thiouracil

## Abstract

The high mortality rate of candidemia and the limited option for the treatment of *Candida* spp. infection have been driving the search for new molecules with antifungal property. In this context, coordination complexes of metal ions and ligands appear to be important. Therefore, this study aimed to synthesize two new copper(II) complexes with 2-thiouracil and 6-methyl-2-thiouracil ligands and to evaluate their mutagenic potential and antifungal activity against *Candida*. The complexes were synthesized and characterized by infrared vibrational spectroscopy, CHN elemental analysis, UV-Vis experiments and ESI-HRMS spectrometry studies. The antifungal activity was evaluated by broth microdilution against 21 clinical isolates of *Candida* species. The mutagenic potential was evaluated by the Ames test. The complexes were Cu(Bipy)Cl_2_(thiouracil) (Complex **1**) and Cu(Bipy)Cl_2_(6-methylthiouracil) (Complex **2**). Complex **1** showed fungicidal and fungistatic activities against all isolates. Furthermore, the Minimum Inhibitory Concentration (MIC) from 31 to 125 µg/mL and inhibition percentage of 9.9% against the biofilms of *C. krusei* and *C. glabrata* were demonstrated. At the concentrations tested, complex **1** exhibited no mutagenic potential. Complex **2** and the free ligands exhibited no antifungal activity at the concentrations evaluated. Since complex **1** presented antifungal activity against all the tested isolates and no mutagenic potential, it could be proposed as a potential new drug for anti-*Candida* therapy.

## 1. Introduction

*Candida* species are one of the most important opportunistic fungal pathogens worldwide. These yeasts are part of the normal microbiota in the human gastrointestinal, respiratory, and reproductive tracts, as well as oral cavity and skin. However, the occurrence of underlying diseases, the use of certain medications, and invasive procedures can cause the multiplication of these yeasts and result in an infectious process [1]. Consequently, *Candida* spp. can infect and cause diseases ranging from mucosal and skin lesions to invasive candidiasis [2]. Literature shows that opportunistic infection of *Candida* species has been related to the increased case of mortality and morbidity in hospitalized patients in recent years [1,3].

In addition to the predisposing factors of the host, the pathogenicity of *Candida* species is affected by their virulence factors, such as the polymorphism and production of tissue-damaging hydrolytic enzymes, such as proteases, phospholipases, and hemolysins, as well as the expression of adhesins, which are related to the biofilm formation (on host tissues or on medical device surfaces) [4]. Studies have showed that most *Candida* infections are associated with biofilm formation [5,6]. This effect contributes to the increase of resistance due to the antifungals’ difficulty in penetrating the sites of infection [7], causing recurrent or chronic infections [8].

Current antifungal therapy is limited to the use of four classes of antifungal agents, namely azoles, polyenes, echinocandins and pyrimidine analogs, which are used orally, topically, and intravenously [9], with the last ones not commercialized in some countries such as Brazil. Some characteristics of these drugs restrict their use in prophylaxis, such as their fungistatic activity, which extends the required time of treatment, high toxicity, and non-specific interaction with other drugs, which increases their side effects [10]. Therefore, the pharmacokinetics of these drugs needs to be improved. 

Recent years have shown increasing number of researches on the development of new antifungal molecules [11,12,13,14] with better bioavailability, safety, and more effective mechanisms of action for the treatment of infections. The coordination of metallic ions with ligands to obtain liposoluble complexes and carriers of biologically active molecules that can improve the antifungal activity [15].

Research on the chemical behavior of thiopyrimidines, such as thiouracils, is of great interest because of the various physiological roles they play [16]. This class of compounds exhibits a variety of bioactivities, including antimicrobial, antiviral, antitumor, antioxidant, and anti-inflammatory actions [17,18]. Thiouracil ligands are N, O, S donor ligands, which are efficient for use in metalation because of the varied possibilities of their coordination with the metallic centers. Copper is an essential trace element of vital physiological functions in humans. Its biological properties stimulate the development of bioactive coordination complexes [19]. Complexes composed of Cu^2+^, Zn^2+^, Cd^2+^, or Hg^2+^ and thiouracil ligands with antimicrobial properties have been described by Kamalakannan et al. [20]. The coordination of bioactive ligands with metal ions to potentiate their biological activities is a strategy widely used in the synthesis of new antifungal drug candidates.

Besides good therapeutic potential, this process is expected to exhibit little to no adverse reactions owing to its low toxicity [21]. Thus, evaluation of the mutagenic potential of the new compounds is relevant in their development as a single drug or in synergism with conventional antifungal agents. In this context, two new copper(II) complexes with 2-thiouracil ligands were synthesized and characterized to evaluate their antifungal activity against 21 clinical isolates of *Candida* species, as well as to evaluate their mutagenic potentials.

## 2. Results

### 2.1. Chemistry

#### 2.1.1. Syntheses of Complex

The preparation of the promising complexes was performed including a modification in the synthetic protocols already published in the literature [22,23]. Reactions of copper(II) chloride, 2,2′-bipyridine, and the thiouracil linkers at 1:1:1 molar ratios in MeOH/THF medium resulted in two promising coordination complexes with the compositions [CuCl_2_(Bipy)(L_1_)], wherein L_1_ = 2-Thiouracil; and [CuCl_2_(Bipy)(L_2_)], wherein L_2_ = 6-methyl-2-thiouracil. Ligand-binder complexes were obtained as an amorphous solid of bluish-green coloration, which have presented different melting points than the starting materials. Their CHN elemental analysis showed consistency between theoretical and experimental data, implying that the complexes obtained were of adequate degree of purity. The results were consistent with the C_14_H_12_CuN_4_Cl_2_SO, C_15_H_15_CuN_4_C_l2_SO molecular formulas, wherein the complexes are in their neutral form.

#### 2.1.2. Vibrational Spectroscopy

Several published studies [16,24,25,26] were used to attribute the main vibrational modes of the complexes (Table 1). The infrared spectra of the complexes **1** and **2** were analyzed in comparison with that of 2-thiouracil, 6-methyl-2-thiouracil, and 2,2′-bipyridine ligands. The ν (N-H) stretching that appears in the region of 3110 to 3006 cm^−1^ in the free ligands, exhibited a shift to larger wavenumbers in the spectra of the complexes, which indicated the coordination of the N-H group to the metallic center. In Complex **1** the band ν (C=O) was shifted to the region with lower energy (1683 cm^−1^) than those of the free ligands (1707 cm^−1^), which can be attributed to the weakening of the C=O group due to the coordination of the oxygen atom to the metallic center. In Complex **2**, the C=O, C=C, and thioamide I bands were superimposed, having a relatively broad band with two IR absorption peaks, namely 1636 and 1557 cm^−1^.

**Figure 1:**
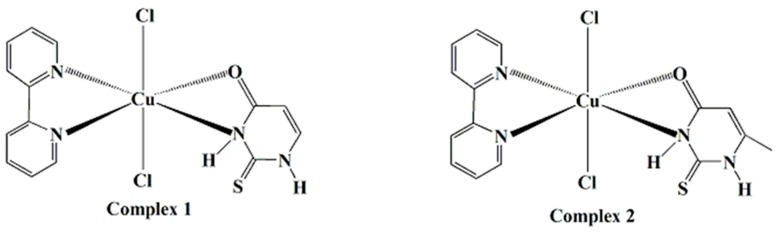
Proposed structures for the complexes **1** and **2**.

After the formation of the complex, the complexes’ spectra presented three distinct bands in this region, namely 1656, 1604, and 1568 cm^−1^. The unfolding of the bands and their displacement to higher energies may be related to the coordination of the C=O group with the metallic center. This coordination is probably caused by the high affinity of copper(II) atom to N and O atoms, and its hard bases, than to the sulfur atom. Both complexes showed no significant shift in the bands related to the thioamides III and IV, compared with those of the free binders, suggesting that the C=S group was not part of the coordination sphere of the metal center. New bands corresponding to the stretches of the 2,2′-bipyridine rings were observed in the spectra of complexes **1** and **2**. Two low intensity absorption peaks were observed in the region of 3084-3034 cm^−1^ corresponding to the ν-(CH)_Ar_ bipyridine ring. The bands were at 1444 cm^−1^ and 1441 cm^−1^ for complexes **1** and **2**, respectively, which were attributed to the vibrations of the pyridine ring. A thin band of average intensity appeared at approximately 770 cm^−1^ in the IR spectra of the complexes and was related to the C-H angular deformation of the aromatic rings in the 2,2′-bipyridine ligand. The stretching band ν (C=N + C=C) of the pyridine ring absorbed energy in the same IR region as the bands of thioamide groups. Infrared absorption spectroscopy and elemental analysis of CHN suggested that the synthesized complexes were presented as neutral complexes with the formulas [CuCl_2_(Bipy)(L_1_)] and [CuCl_2_(Bipy)(L_2_)] (Figure 1).

The two promising copper(II) complexes with thiouracil ligands were formed by the coordination of the respective ligands to the metal ion in bidentate manner by the N and O atoms. Concomitantly, the 2,2′-bipyridine ligands was coordinated bidentately by the N atoms of the pyridine rings to the metallic center, completing the coordination sphere of two Cl atoms and conferring a slightly octahedral coordination environment to the Cu^2+^ ion.

#### 2.1.3. ESI-HRMS Spectrometry

ESI-HRMS spectrometry experiments were performed to study the composition of the complexes **1** and **2**. The HRMS spectra for both complexes can be found in Figures S1 and S2. In spite of the ESI-HRMS experiments did not show the expected molecular ion peak *m*/*z* 416.9405 for complex **1** and *m*/*z* 430.9561 for complex **2**, (it is worth mentioning that copper complexes could undergo redox reactions during ionization, which in turn contributes to the collapse of their structures [27,28,29] we could detect that our complexes collapse in a few fragments and most of them presented the copper ion bonded to the tiouracile ligands or fragments thereof. The main peaks observed for the complex **1** (*m*/*z* = 157.0759, 260.0270, 375.0667) and for the complex **2** (*m*/*z* = 157.0760, 237.0071, 375.0648) agree with the proposed structures. As can be seen from Figures S1 and S2 the peak at 157.0759 is related to the pyridine coligand and the peaks at 260.0270 and 375.0648 are related to the species containing copper (copper presents natural isotopic abundance of ^63^Cu = 0.6915 (15) and ^65^Cu = 0.3085 (15)). The isotopic abundance of the copper ion was observed in the main peaks containing the metal-organic fragments and has been emphasized in Figures S1 and S2.

### 2.2. UV-Vis Studies and Stability Evaluation

**Figure 2:**
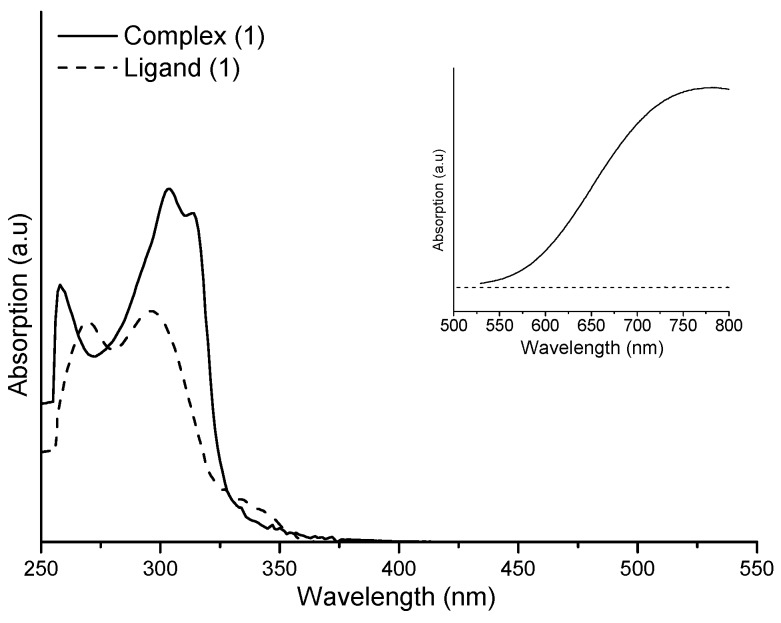
Absorption spectra of the complex **1** and free ligand measured at 298 K using the same concentration (1 × 10^−5^ M H_2_O/DMSO (95/05%).

**Figure 3:**
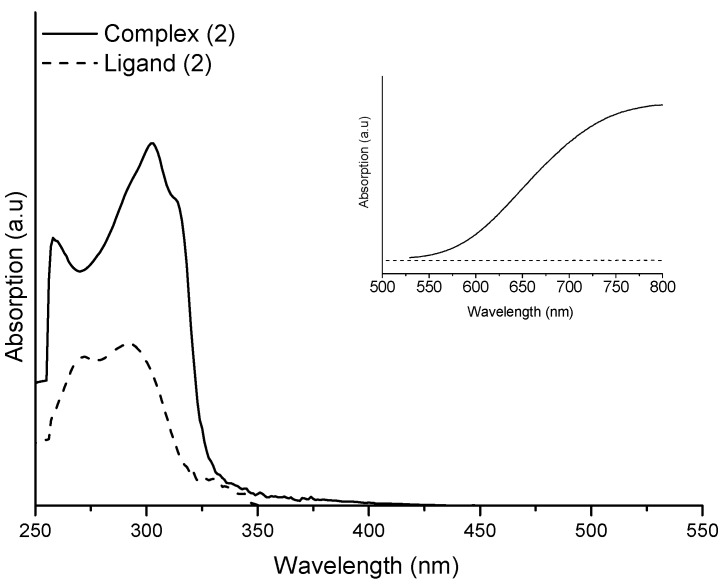
Absorption spectra of the complex **2** and free ligand measured at 298 K using the same concentration (1 × 10^−5^ M H_2_O/DMSO (95/05%).

To study the photophysical properties and the solution stability of the prepared complexes we decided performing UV-Vis experiments using the same experimental conditions used in the biological assays. The UV-Vis spectra of the prepared complexes and free ligands are shown in Figure 2 and Figure 3.

It can be seen from Figure 2 and Figure 3 an hyperchromic effect from the free ligand to complexes **1** and **2**. This behavior has been expected after metalation of the ligand to the Cu metallic atom and it is an experimental evidence for the complexes formation. The light green color presented by the complexes’ solution (Figure S3) is related to the metal centered d → d Cu^II^(d^9^) transitions that appeared as a broad band from 550 to 800 nm in the spectra of the complexes **1** and **2** (insets graph in the top of the figures). It is worth mentioning that these electronic transitions are absent in the spectra of the ligands. Two absorption bands centered at ca. 320 and 260 nm were observed in the UV-Vis spectra of the complexes **1** and **2** and at ca. 290 and 275 nm for the ligands. The band centered at ca. 290 nm and 275 nm for the free ligands can be assigned to the IL (intra ligand) electronic transitions of the type (O, S)n → π* and π → π* respectively. After complexation of the ligands to the copper(II) atom, the band centered at ca. 290 nm presented a bathochromic effect (30 nm red shifted) appearing at ca. 320 nm in the UV-Vis spectra of the complexes. It is related to the appearance of LMCT (ligand to metal charge transfer) electronic transitions which should be present after complexation of ligands in transition metals [23]. The π → π* electronic transitions related to the bipyridine coligand and aromatic system of the thiouracile ring that appeared centered at ca. 275 nm for both ligands presented an hypsochromic effect (blue shifted 15 nm) from ligands to complexes **1** and **2**. The hypsochromic effect on the π → π* electronic transitions is also expected after coordination of ligands to the copper atom and this behavior reinforces the experimental evidence of the complexes formation. To determine the stability of the complexes **1** and **2** in solution (H_2_O/DMSO), we decided to perform the same UV-Vis experiment 36 h after preparing the solutions. The Figures S4 and S5 are shown the absorption experiments of the complexes **1**, **2** and respective ligands. It can be seen from Figures S4 and S5 that the absorption profile of the prepared complexes **1** and **2** did not show a difference after 36 h and the same spectral behavior was observed. Additionally, any feature of free ligand was observed after 36 h in the spectra of the complexes **1** and **2**, in this sense, it is reasonable to interpret that the complexes are maintaining their structures in solution and the disruption of metal-ligand bonds were not observed in the light of the UV-Vis experiments.

### 2.3. Antifungal Activity

The antifungal activity of the complex **1** [CuCl_2_(Bipy)(L_1_)], 2 [CuCl_2_(Bipy)(L_2_)], free ligands (2-thiouracil and 6-methyl-2-thiouracil) and copper(II) chloride in planktonic cells, were evaluated against isolates of *Candida* species. Regarding complex **1**, the Minimum Inhibitory Concentration (MIC) values ranged from 31.25 to 125 μg/mL and the Minimum Fungicidal Concentration (MFC) from 31.25 to 250 μg/mL. *C*. *krusei* was the most sensitive isolate, with MIC value of 31.25 μg/mL (Table 2). Complex **2** and the free ligands exhibited no antifungal activity at the concentrations evaluated. The copper(II) chloride exhibited a MIC of 1000 μg/mL for all isolates (Table S1), this is an experimental evidence that the antifungal activity of complex **1** should be related to the synergistic effect between thiouracil ligand and the copper ion in the complex structure.

### 2.4. Biofilm Formation

**Figure 4:**
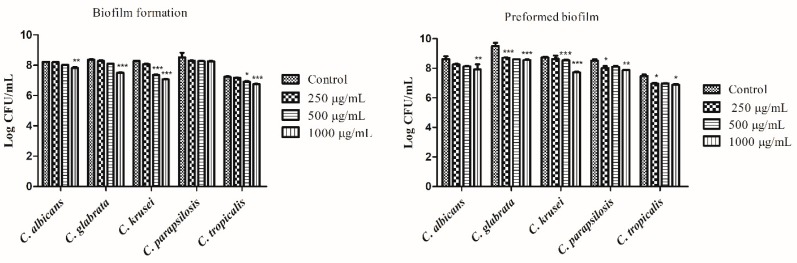
Effect of complex **1** [CuCl_2_(Bipy)(L_1_)] during biofilm formation and on preformed biofilms of *Candida* species. * *p* <0.05; ** *p* <0.01; *** *p* <0.001 (ANOVA).

Based on the results of the antifungal activity test on planktonic cells, one isolate of each species—*C. albicans* CA1, *C. glabrata* CG1, *C. krusei* CK1, *C. parapsilosis* CP1 and *C. tropicalis* CT1—was randomly selected for the examination of Complex **1** effect on both formed and preformed biofilm. Complex **1** significantly reduced the formation of biofilm by some *Candida* species (Figure 4). Complex **1** at the concentration of 1000 μg/mL reduced the number of cultivable cells of all species tested, with *C. krusei* (~1.2 log) and *C. glabrata* (~0.8 log) showing the most reduction.

The effect of complex **1** observed on preformed *Candida* biofilms was similar to those found in the case of *C. krusei* and *C. glabrata* biofilm formation, showing the greatest reduction in the number of cultivable cells (~1.0 log and 0.9 log, respectively). Thus, in both assays, Complex **1** showed a percentage inhibition of approximately 9.9% against *C. Krusei* and *C. glabrata* biofilm formations.

### 2.5. Mutagenic Activity

The new copper(II) complex was also evaluated for its mutagenic potential by the Ames test using *Salmonella typhimurium* (Kado method). The results are shown in Table 3. Based on the number of revertant colonies per plate, complex **1** exhibited no mutagenic potential on TA98 and TA100 cell lines in the presence and absence of metabolic activation. The mutagenic potential index was lower than 2 and did not suggest a dose-response relationship.

## 3. Discussion

Opportunistic fungal infection by *Candida* species has been a recurrent health problem, against which the search for new therapeutic agents has been intensifying in recent years [1]. In this study, the synthesis of two potential antifungal coordination complexes, which consisted of copper(II) center with 2-thiouracil (complex **1**) and 6-methyl-thiouracil (complex **2**) ligands, was performed.

Complex **1** showed antifungal activity against 21 clinical isolates of *Candida* species which are common causes of fungal infection. The lowest MIC value of Complex **1** was against *C. krusei* (31.25 µg/mL), in which the highest percentage of biofilm inhibition was observed. This finding could be significant because as *C. krusei* is intrinsically resistant to fluconazole, and its therapy option is restricted to the use of equinicandins and amphotericin B [30], which have been associated with increased episodes of candidemia and invasive candidiasis [31]. The severe clinical cases of *C. krusei*, coupled with the lack of available antifungal treatment against it, resulted in high mortality rate. Complex **1** also showed inhibitory activity against planktonic and sessile cells (in the biofilm) of *C. glabrata*, which was resistant to fluconazole in a dose-dependent manner. Surveillance studies have reported on the resistance of *C. glabrata* isolates to triazoles, and, more recently, to echinocandins, thus characterizing this species as multi-drug resistant [32].

Regarding the effect of Complex **1** on yeast viability, the complex showed fungicidal and fungistatic activities against all isolates. This is a relevant finding, since azoles, such as fluconazole, has only fungistatic activity. These data suggested that the fungicidal activity of the complex also influenced its ability to inhibit biofilm formation and destroy preformed biofilm. The ability to form biofilm is one of the virulence factors increasing the pathogenicity of *Candida*. The complex structure and architecture of the sessile cells in a biofilm increased their resistance to antifungal drugs [33], with several studies reporting that sessile cells in biofilms have greater resistance than do planktonic cells. According to some studies, the necessary MIC of antimicrobial agents for the reduction in biofilm formation is 2 to 1000 times greater than that for planktonic cells [33,34,35]. It is important that in this study, Compound 1 was able to reduce the number of viable cells in biofilms at a concentration of approximately 16-fold the MIC, namely 500 μg/mL for *C. krusei* and 1000 μg/mL for the other species.

The data in this study suggested that the coordination of the 2-thiouracil ligand to the copper(II) metallic center (Complex **1**) potentiated the antifungal activity of the same, since in an equimolar concentration the copper(II) chloride exhibited MIC (1000 μg/mL). Mohamed et al. [36,37] and Masoud et al. [38] also reported the antifungal activity of copper complexes with 2-thiouracil binder, which may result from the increased liposolubility of the complex and the toxicity of copper on microorganisms, which causes membrane damage and leads to cell death [39,40]. It was verified that the alteration of ligand increased its antifungal activity. Complex **2** had no effect on the isolates tested. Since the structural difference between the two compounds is in the presence of the methyl substituent at the 6-position of the thiopyrimidine ring, it is thought that the substituent interfered with the liposolubility and consequently the antifungal activity of Complex **2**.

For pharmaceutical application, Complex **1** was also evaluated for its mutagenic potential by the Ames test using TA98 (*hisD305*2/*rfa*/*urvB*/*pKM101*/ApR) and TA100 (*hisG46*/*rfa*/*ΔuvrB*/*pKM101*/ApR), which allowed us to examine frameshift and base pair substitution mutations, respectively. Complex **1** did not induce these types of mutations at the concentrations tested. Understanding the genotoxic characteristics of a new complex is key to further pharmaceutical development. According to the International Conference on Harmonization of Technical Requirements for Registration of Pharmaceutical Products of the Food and Drug Administration (FDA) of the United States, the Ames test is part of a series of standardized tests to evaluate the genotoxicity of new drugs, which is necessary as studies have reported rodent-carcinogenic drugs by the reverse mutation test using lineages of *S. typhimurium* [41]. The Ames test also allows direct and indirect evaluation of the mutagenic potential of new compounds owing to the use of S9 microsomal fraction that simulates a metabolism process in mammals, guaranteeing high sensitivity of the test [42]. Thus, the use of these tests is of extreme importance for research and development of new complexes as therapeutic agents.

## 4. Materials and Methods

### 4.1. Chemical and Measurements

The compounds 2-thiouracil, 6-methyl-2-thiouracil, 2,2′-bipyridine, and copper(II) chloride employed in the complex syntheses were purchased from Sigma Aldrich (St. Louis, MO, USA). Solvents (grade PA) were commercially obtained and used without further purification. CHN elemental analyses were performed using a Perkin-Elmer 2400 Analyzer (Perkin-Elmer, Waltham, MA, USA). FT-IR spectra were obtained by a JASCO-4100 spectrophotometer (Jasco, Easton, MD, USA) in the spectral window of 4000–400 cm^−1^, using sample dispersion in KBr. Melting point values were determined on a DF-3600 Instruterm apparatus (Instruterm, São Paulo, SP, Brazil). UV-Vis analyses were performed in the same biological experimental conditions on double beam PerkinElmer Spectrophotometer Lambda 650S using quartz cuvettes of 1 cm optical path. HRMS was performed on a Bruker Mass Spectrometer IES-Q-QTOF-micro TOF III (Bruker Daltonics) in the positive mode (*m*/*z* 120–1200), quadrupole Ion energy 5.0 eV and cell collision energy of 10 eV. The samples were prepared using 0.0001 g/mL (water) and 0.1% of formic acid.

### 4.2. Preparation of Complexes

**Scheme 1:**
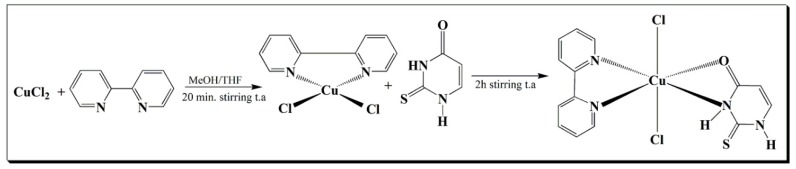
Reaction route for the obtention of the Complex **1**.

Complex **1**: According to the scheme below (Scheme 1), 0.0134 g (0.1 mmol) copper chloride was dissolved in 6 mL of THF/MeOH (1:1, *v*/*v*). A light-green solid was obtained in 67% yield (Figure S6). The same procedure was used for the synthesis of Complex **2** using 6-methyl-2-thiouracil ligand and Complex **2** was obtained in 72% yield (Figure S7). The same procedure was used for the synthesis of Complex **2** using 6-methyl-2-thiouracil ligand. The preparation was performed including a modification in the synthetic protocols already published in the literature [22,23].

Complex **1** [CuCl_2_(Bipy)(L_1_)]: Elemental analysis of CHN theoretical to C_14_H_12_CuN_4_Cl_2_SO, MW = 418.78 g/mol: C = 40.14%, H = 2.89%, N = 13.38%. Experimental: C = 40.17%, H = 2.92%, N = 13.41%. IR (KBr, *ν*/cm^–1^): 3109–3089 (*ν*(N-H)), 3051–3035 (*ν*(C-H)_AR._), 2928 (*ν*(C-H)), 1683, 1602(*ν*(C=O), *ν*(C=C)), 1557 (thiomide I: *δ*(NH) + *ν*(C-N)), 1444 (ν(ring) + *δ*(C-H)_bipy_), 1240 (amide III: *ν*(C-N) + *δ*(C-N-H)), 1207 (thiomide II: *ν*(C-N) + *δ*(NH) + *δ*(C-H)) 1173, 1158 (thiomide III: *ν*(C-N) + *ν*(C=S)), 831 (thiomide IV: *ν*(C=S)), 777 (*δ*(C-H)_bipy_). Melting point: 240 °C.

Complex **2** [CuCl_2_(Bipy)(L_2_)]: Elemental analysis of CHN theoretical to C_15_H_14_CuN_4_Cl_2_SO, MM = 432.81 g/mol: C = 41.63%, H = 3.26%, N = 12.94%. Experimental: C = 41.57%, H = 3.27%, N = 12.97%. IR (KBr, *ν*/cm^–1^): 3109–3090 (*ν*(N-H)), 3051–3034 (*ν*(C-H)_AR._), 2930 (*ν*(C-H)), 1656, 1604(*ν*(C=O), *ν*(C=C)), 1568 (thiomide I: *δ*(NH) + *ν*(C-N)), 1441 (ν(ring) + *δ*(C-H)_bipy_), 1245 (amide III: *ν*(C-N) + *δ*(C-N-H)), 1193 (thiomide II: *ν*(C-N) + *δ*(NH) + *δ*(C-H)) 1167 (thiomide III: *ν*(C-N) + *ν*(C=S)), 835 (thiomide IV: *ν*(C=S)), 777 (*δ*(C-H)_bipy_). Melting point: 290 °C.

### 4.3. Microorganisms and Culture Conditions

To evaluate the antifungal activity of the copper(II) complexes, 21 clinical isolates of *Candida* species collected from different body parts were obtained from the collection of the Laboratory of Applied Microbiology, Universidade Federal da Grande Dourados, Brazil. The isolates were identified as *C. albicans* (ten isolates), *C. glabrata* (six isolates), *C. krusei* (one isolate), *C. parapsilosis* (three isolates), and *C. tropicalis* (one isolate).

All species were stored at −20 ± 2 °C in Sabouraud Dextrose Broth (SDB, HiMedia Laboratories, Mumbai, India) with 20% (*v*/*v*) glycerol. Prior to each assay, species were subcultured from the frozen stock suspension onto Sabouraud Dextrose Agar (SDA, HiMedia) plates. The plates were incubated overnight at 35 °C. A pool of growing colonies was subcultured in CHROMagar *Candida^®^* (Difco, Tlalnepantla, Estado de Mexico, Mexico) to investigate the purity of the culture and color of the colony. Yeasts that grew in the differential selective medium were identified according to conventional methodology [43].

### 4.4. Antifungal Activity Screening

The screening of the antifungal activity of Complexes 1 and 2 was performed by broth microdilution in 96-well polystyrene plates according to the standard M27-A3 of the Clinical and Laboratory Standards Institute (CLSI) [44].

Microplates containing the serial dilutions of the complexes with concentrations ranging from 1.9 to 1000 μg/mL were inoculated with 100 μL suspension containing 2.5 × 10^3^ CFU/mL of either yeast in RPMI 1640 medium (Sigma Aldrich). Fluconazole and Amphotericin B were used as reference drugs. The plates were incubated at 35 °C for 48 h. The MIC was considered as the lowest complex concentration which showed no visible growth of microorganisms after incubation.

The number of viable cells was calculated by determining the Colony Forming Units (CFUs) following serial dilutions and incubation in SDA for 24 h. The MFC was defined as the lowest concentration of complex that yielded no fungal growth.

As defined by the CLSI, negative controls (medium only) and positive controls (medium and yeast) were used in each test. The cut-off levels of susceptibility to fluconazole (Sigma Aldrich) were utilized according to the CLSI [44] to identify species as susceptible (S ≤ 8 µg/mL), dose-dependent susceptible (DDS = 16–32 µg/mL), and resistant (R ≥ 64 µg/mL). This document did not consider amphotericin B, so the susceptibility references levels established by Yang et al. [45] were used: values of MIC ≤ 1 µg/mL indicated the yeast was susceptible, and levels ≥2 µg/mL indicated the yeast was resistant. The subsequent tests were performed based on the results obtained in this screening process.

### 4.5. Biofilm Formation

Considering the antifungal activity of the Complex **1**, its effect was evaluated on the biofilm and preformed biofilm of *Candida* species. Species that are particularly sensitive to the complex were selected, namely *C. albicans*, *C. glabrata*, *C. krusei*, *C. parapsilosis*, and *C. tropicalis*. The concentrations of the compound used in this test were based on the results of the antifungal susceptibility test (Section 4.4). The assays were performed according to a previous study [46], with several adaptations.

#### 4.5.1. Effect of the Complex **1** on Biofilm Formation

To evaluate the effect of Complex **1** on biofilm formation, *Candida* species were inoculated in SDB and incubated for 18 h at 35 °C under 120 rpm shaking. Afterwards, the yeasts were centrifuged at 3000 *g* for 10 min and washed three times with phosphate buffered saline (PBS), pH 7. One hundred microliter of a standard inoculum suspension (1 × 10^7^ cells/mL) was inoculated simultaneously to 100 μL complex diluted in SDB at the concentrations of 250, 500, and 1000 µg/mL. The inoculums were plated in wells and incubated under shaking (120 rpm/min) for 48 h at 35 °C.

Following incubation, the content of each well was removed. The remaining biofilms were washed three times with 200 μL PBS to remove weakly adhered cells and each well was scraped vigorously with a pipette tip to collect the biofilm.

The effect of Complex **1** on biofilm formation was examined by counting the viable cells in the biofilms. Serial dilutions of the suspensions were plated on SDA and incubated for 24 h at 35 °C. The total number of CFUs per unit area (log CFU/mL) of the each well was counted.

#### 4.5.2. Effect of the Complex **1** on Preformed Biofilms

To evaluate the effect of Complex **1** on preformed biofilms, 200 μL standard inoculum suspension (1 × 10^7^ cells/mL) prepared in SDB was added to a 96-well microplate. The plates were incubated in an incubator with orbital shaking at 120 rpm/min for 48 h. Subsequently, the medium was carefully aspirated and washed three times with PBS to remove the weakly adhered cells. Complex **1** at concentrations 250, 500, and 1000 µg/mL diluted in SDB was added to the wells. After 24 h incubation at 35 °C, the content of each well was discarded, and the plates were washed three times with PBS to remove non-adherent cells and each well was scraped vigorously with a pipette tip. To evaluate the effect of the complex, the CFU value of each suspension was quantified and presented as log CFU/mL.

### 4.6. Mutagenic Activity

The evaluation of the mutagenic potential of Complex **1** was performed by Ames test as described by Kado et al. [47]. The standardized TA98 and TA100 *Salmonella typhimurium* strains were used at a concentration of 1 × 10^8^ cells/mL.

The assay was performed in the presence and absence of metabolic activation of S9, which was prepared from the liver of Sprague-Dawley rats treated with polychlorinated biphenyl aroclor 1254 mixture (500 mg/kg). The S9 fraction was acquired from Molecular Toxicology Inc. (Boone, NC, USA) and freshly prepared before each test. Fifty microliter of 0.2 M phosphate buffer or S9 fraction, 5 μL of complex (15, 50, 150, 500, 1500, and 5000 μg/plate), and 50 μL of bacterial suspension were added to test tubes. The tubes were preincubated for 90 min at 37 °C. Soon after, 2 mL Top Agar (0.6% agar, 0.6% NaCl, 0.05 mM L-histidine, 0.05 mM biotin, pH 7.4, 45 °C) was added and then the mixture was poured into Minimal Agar plates (1.5% agar, Voguel-Boner medium, and 10% glucose solution). The plates were incubated at 37 °C for 48–66 h, after which the His + revertant colonies were counted.

The positive controls used in the tests without metabolic activation were: 4-nitrophenylenediamine (NPD) (10 μg/plate) for the TA98 line and sodium azide (2.5 μg/plate) for the TA100 lineage. In the metabolic activation assays, 2-aminoanthracene (2-ANTR) (0.63 μg/plate) was used for both strains. Dimethylsulfoxide (DMSO) was used as the negative control. All reagents used as positive and negative controls were purchased from Sigma Aldrich. The assays were performed in triplicate for each concentration of the extract.

### 4.7. Statistical Analysis

The results of the biofilm assays were analyzed by two-way ANOVA and Bonferroni post-test using the Graph Pad Prism 7.0 software. The Ames test data were analyzed using the Salanal statistical program version 1.0 (U.S. Environmental Protection Agency, Monitoring Systems Laboratory, Las Vegas, NV, USA from Research Triangle Institute, RTP, Research Triangle Park, NC, USA), adopting the model used by Bernstein et al. [48].

## 5. Conclusions

We have prepared and characterized two new copper(II) complexes, which were evaluated for their antifungal and mutagenic potential. Complex **1** [CuCl_2_(Bipy)(L_1_)] exhibited fungicidal activity against all *Candida* isolates tested, showing antifungal activity against both planktonic and sessile cells. *Candida krusei* was the most sensitive species to the complex. Complex **2** [CuCl_2_(Bipy)(L_2_)] and the free ligands exhibited no antifungal activity at the concentrations evaluated. Considering that complex **1** showed no mutagenic potential at the concentrations tested, we propose complex **1** as a new drug in the field of anti-*Candida* therapy.

## Figures and Tables

**Table 1:** Modes and vibrational frequencies of Bipyridine, ligands L_1_ and L_2_, and complexes **1** and **2**.

Frequency Vibrations (cm^−1^)					
	Ligand (1)	Ligand (2)	Bipy	Complex (1)	Complex (2)
ν (N-H)	3086-3046	3110-3006	-----	3109-3089	3109-3090
ν(C=O, C=C)	1707, 1616	1636, -----	-----	1683, 1602	1656, 1604
thioamide I	1567	1557	-----	1557	1568
thioamide II	1216	1200	-----	1207	1193
thioamide III	1173	1167	-----	1173	1167
thioamide IV	833	837	-----	832	835
ν(C-H)_Ar_	-----	-----	3084-3051	3051-3035	3051-3034
ν(C-H)	2921	2928	-----	2928	2930
ν((ring) + *δ*(C-H))	-----	-----	1453	1444	1441
ν(C=N + C-N)	-----	------	1583	-----	-----
δ(C-H)_Ar_	-----	-----	754	771	777

**Table 2:** Anti-*Candida* activity of the complex **1** [CuCl_2_(Bipy)(L_1_)] in planktonic cells by microdilution in broth technique (µg/mL).

Isolate	Source	MIC ^a^	MFC ^b^	FLC ^c^	AmB ^d^
*C. albicans* CA1	Sputum	62.5	62.5	0.5	0.5
*C. albicans* CA2	Sputum	62.5	125	0.25	0.25
*C. albicans* CA3	Vaginal	62.5	62.5	0.5	0.5
*C. albicans* CA4	Vaginal	62.5	62.5	0.5	0.5
*C. albicans* CA5	Vaginal	62.5	62.5	0.5	0.5
*C. albicans* CA6	Vaginal	62.5	62.5	0.5	0.5
*C. albicans* CA7	Nasal swab	125	250	0.25	0.5
*C. albicans* CA8	Urine	125	125	0.25	0.5
*C. albicans* CA9	Vaginal	125	125	0.25	0.5
*C. albicans* CA10	Vaginal	125	125	0.5	0.5
*C. glabrata* CG1	Urine	62.5	62.5	16 ^e^	0.03
*C. glabrata* CG2	Urine	62.5	62.5	1	0.03
*C. glabrata* CG3	Urine	62.5	125	16 ^e^	0.5
*C. glabrata* CG4	Urine	62.5	125	1	0.03
*C. glabrata* CG5	Urine	62.5	62.5	32 ^e^	0.03
*C. glabrata* CG6	Urine	125	250	16 ^e^	0.5
*C. krusei* CK1	Rectal swab	31.25	31.25	0.5	0.03
*C. parapsilosis* CP1	Blood	62.5	125	0.25	0.25
*C. parapsilosis* CP2	Catheter tip	125	125	0.25	0.03
*C. parapsilosis* CP3	Urine	125	250	0.25	0.03
*C. tropicalis* CT1	Sputum	62.5	125	2	0.5

^a^ MIC: Minimum Inhibitory Concentration (µg/mL); ^b^ MFC: Minimum Fungicidal Concentration (µg/mL); ^c^ FLC: Fluconazole; ^d^ Amphotericin B; ^e^ Susceptible Dose-Dependent (SDD) (µg/mL).

**Table 3:** Mutagenic activity expressed by the mean of revertant/plate index of mutagenicity (IM) of complex **1** [CuCl_2_(Bipy)(L_1_)] in the TA98 and TA100 strains of *S. typhimurium* in the presence (+S9) and absence (−S9) of activation metabolic.

Treatment (µg/plate)	TA98		TA100	
	−S9	+S9	−S9	+S9
0.0 ^a^	42 ± 2	15.00 ± 2	192 ± 6	191 ± 8
15	31 ± 1 (0, 7)	15 ± 2 (1, 0)	169 ± 9 (0, 9)	176 ± 8 (0, 9)
50	31 ± 4 (0, 7)	16 ± 1 (1, 0)	229 ± 7 (1, 2) *	201 ± 7 (1, 0)
150	34 ± 3 (0, 8)	17 ± 1 (1, 1)	265 ± 9 (1, 4) **	190 ± 7 (1, 0)
500	36 ± 1 (0, 8)	14 ± 2 (1, 0)	226 ± 6 (1, 2) *	241 ± 6 (1, 2) **
1500	34 ± 2 (0, 8)	16 ± 3 (1, 1)	249 ± 5 (1, 3) **	241 ± 8 (1, 3) **
5000	36 ± 5 (0, 8)	18 ± 2 (1, 2)	286 ± 7 (1, 5) **	238 ± 9 (1, 4) **
C+	246 ± 6 ^b^	227 ± 9 ^c^	990 ± 9 ^d^	979 ± 4 ^c^

^a^ Negative control: DMSO; Positive Control (C+): ^b^ 4-nitro-o-phenylenediamine (10 μg/plate); ^c^ 2-aminoanthracene (1.5 μg/plate); ^d^ Sodium azide (2.5 μg/plate). * *p* < 0.05; ** *p* < 0.01 (ANOVA).

## Supplementary Materials

Supplementary Materials are available online. Table S1: Anti-*Candida* activity of the complex **2** [CuCl_2_Bipy(L_2_)], free ligands (2-thiouracil and 6-methyl-2-thiouracil) and cooper(II) chloride in planktonic cells by microdilution in broth technique (µg/mL). Figure S1: ESI-HRMS spectrum of the complex **1**. Figure S2: ESI-HRMS spectrum of the complex **2**. Figure S3: Cuvettes containing the solutions of the complex **1** (light green) and ligand (colorless). Picture taken during the experiment. Complex **2** and respective ligand have been presented similar behavior. Figure S4: UV-Vis absorption spectra of the complex **1** and respective ligand measured at 298 K. This experiment was performed 36 h after preparing the solutions. Figure S5: UV-Vis absorption spectra of the complex **2** and respective ligand measured at 298 K. This experiment was performed 36 h after preparing the solutions. Figure S6: Green solid of the Complex **1** obtained after filtration. Figure S7: Green solid of the Complex **2** obtained after filtration.
