# Supplementary material for: A Promising Copper(II) Complex as Antifungal and Antibiofilm Drug against Yeast Infection
Source: Molecules. 2018 Jul 26;23(8):1856. doi: 10.3390/molecules23081856 (PMC6222317; doi:10.3390/molecules23081856)
Supplement: Supplementary file 1 [file molecules-23-01856-s001.pdf]

molecules

A Promising Copper(II) Complex as Antifungal and Antibiofilm Drug against Yeast Infection

Fabiana Gomes da Silva Dantas [1], Adriana Araújo de Almeida-Apolonio [2], Renata Pires de Araújo [3], Lis Regiane Vizolli Favarin [4], Pamella Fukuda de Castilho [1], Fernanda de Oliveira Galvão [1], Terezinha Inez Estivalet Svidzinski [5], Gleison Antônio Casagrande [4,*] and Kelly Mari Pires de Oliveira [1,6,*]

[1] Faculty of Health Sciences, Federal University of Grande Dourados, Dourados, MS 79804-970, Brazil; fabianasilva@ufgd.edu.br (F.G.d.S.D.); pamellafcastilho@gmail.com (P.F.d.C.); feergalvao@hotmail.com (F.d.O.G.)
[2] Faculty of Medicine, Federal University of Mato Grosso do Sul, Campo Grande, MS 79070-900, Brazil; aaraujo.a@hotmail.com (A.A.d.A.A)
[3] Faculty of Exact Sciences and Technology, Federal University of Grande Dourados, Dourados, MS 79804-970, Brazil; renataaraujo@ufgd.edu.br (R.P.d.A)
[4] Institute of Chemistry, Federal University of Mato Grosso do Sul, Campo Grande, MS 79074-460, Brazil; lisregiane@hotmail.com (L.R.V.F)
[5] Department of Clinical Analysis and Biomedicine, State University of Maringá, Maringá, PR 87020-900, Brazil; terezinha.svidzinski@gmail.com (T.I.E.S)
[6] Faculty of Biological and Environmental Science, Federal University of Grande Dourados, Dourados, MS 79804-970, Brazil
* Correspondence: gleisoncasag@gmail.com (G.A.C.); kellyoliveira@ufgd.edu.br (K.M.P.d.O.); Tel.: +55-067-3345-3595 (G.A.C.); +55-067-3410-2220 (K.M.P.d.O.)

Received: 09 June 2018; Accepted: 26 June 2018; Published: date

molecules

MDPI

Table S1. Anti-*Candida* activity of the complex 2 [CuCl2Bipy(L2)], free ligands (2-thiouracil and 6-methyl-2-thiouracil) and cooper(II) chloride in planktonic cells by microdilution in broth technique (μg/mL).

Isolate	Source	Complex 2 [$CuCl_2$Bipy(L2)]		2-thiouracil		6-methyl-2-thiouracil		$CuCl_2$		FLC[c]	AmB[d]
		MIC[a]	MFC[b]	MIC[a]	MFC[b]	MIC[a]	MFC[b]	MIC[a]	MFC[b]		
C. albicans CA1	Sputum	>1000	>1000	>1000	>1000	>1000	>1000	1000	1000	0.5	0.5
C. albicans CA2	Sputum	>1000	>1000	>1000	>1000	>1000	>1000	1000	1000	0.25	0.25
C. albicans CA3	Vaginal	>1000	>1000	>1000	>1000	>1000	>1000	1000	1000	0.5	0.5
C. albicans CA4	Vaginal	>1000	>1000	>1000	>1000	>1000	>1000	1000	1000	0.5	0.5
C. albicans CA5	Vaginal	>1000	>1000	>1000	>1000	>1000	>1000	1000	1000	0.5	0.5
C. albicans CA6	Vaginal	>1000	>1000	>1000	>1000	>1000	>1000	1000	1000	0.5	0.5
C. albicans CA7	Nasal swab	>1000	>1000	>1000	>1000	>1000	>1000	1000	1000	0.25	0.5
C. albicans CA8	Urine	>1000	>1000	>1000	>1000	>1000	>1000	1000	1000	0.25	0.5
C. albicans CA9	Vaginal	>1000	>1000	>1000	>1000	>1000	>1000	1000	1000	0.25	0.5
C. albicans CA10	Vaginal	>1000	>1000	>1000	>1000	>1000	>1000	1000	1000	0.5	0.5
C. glabrata CG1	Urine	>1000	>1000	>1000	>1000	>1000	>1000	1000	1000	16[e]	0.03

C. glabrata CG2	Urine	>1000	>1000	>1000	>1000	>1000	>1000	1000	1000	1	0.03
C. glabrata CG3	Urine	>1000	>1000	>1000	>1000	>1000	>1000	1000	1000	16[e]	0.5
C. glabrata CG4	Urine	>1000	>1000	>1000	>1000	>1000	>1000	1000	1000	1	0.03
C. glabrata CG5	Blood	>1000	>1000	>1000	>1000	>1000	>1000	1000	1000	32[e]	0.03
C. glabrata CG6	Urine	>1000	>1000	>1000	>1000	>1000	>1000	1000	1000	16[e]	0.5
C. krusei CK1	Rectal swab	>1000	>1000	>1000	>1000	>1000	>1000	1000	1000	0.5	0.03
C. parapsilosis CP1	Blood	>1000	>1000	>1000	>1000	>1000	>1000	1000	1000	0.25	0.25
C. parapsilosis CP2	Catheter tip	>1000	>1000	>1000	>1000	>1000	>1000	1000	1000	0.25	0.03
C. parapsilosis CP3	Urine	>1000	>1000	>1000	>1000	>1000	>1000	1000	1000	0.25	0.03
C. tropicalis CT1	Sputum	>1000	>1000	>1000	>1000	>1000	>1000	1000	1000	2	0.5

[a]MIC: Minimum Inhibitory Concentration (µg/mL); [b]MFC: Minimum Fungicidal Concentration (µg/mL); [c]FLC: Fluconazole (µg/mL); AmB: [d]Amphotericin B (µg/mL); [e]Susceptible Dose-Dependent (SDD) (µg/mL).

Complex 1

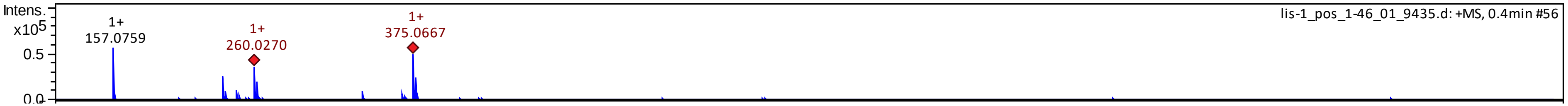

Full spectrum

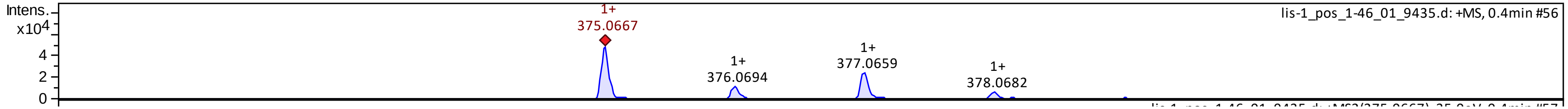

Zoom in 375.0667 emphasis on the isotopic distribution of copper.

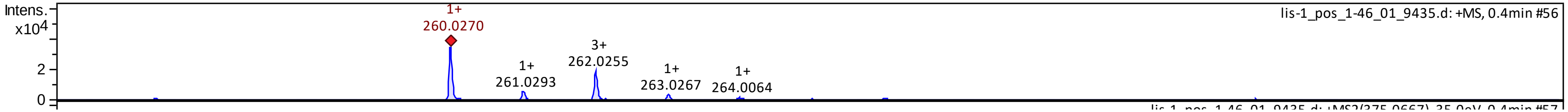

Zoom in 260.0270 emphasis on the isotopic distribution of copper.

Figure S1. ESI-HRMS spectrum of the complex 1.

Complex 2

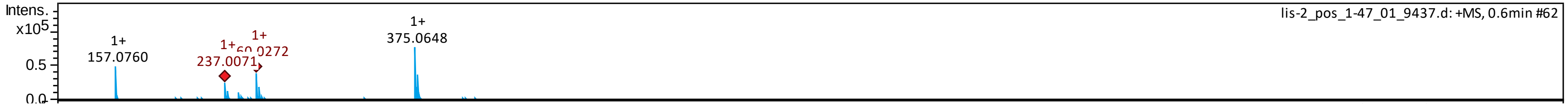

Full spectrum

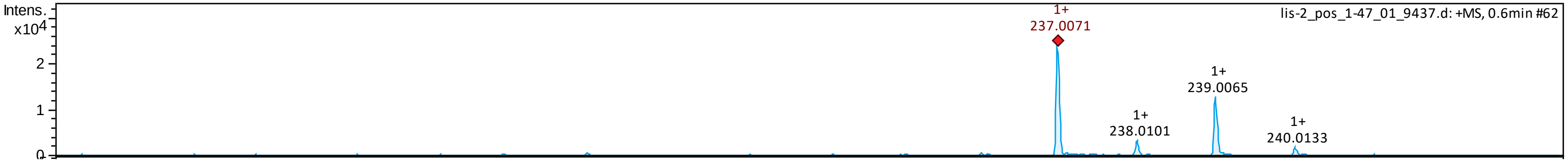

Zoom in 237.0071 emphasis on the isotopic distribution of copper.

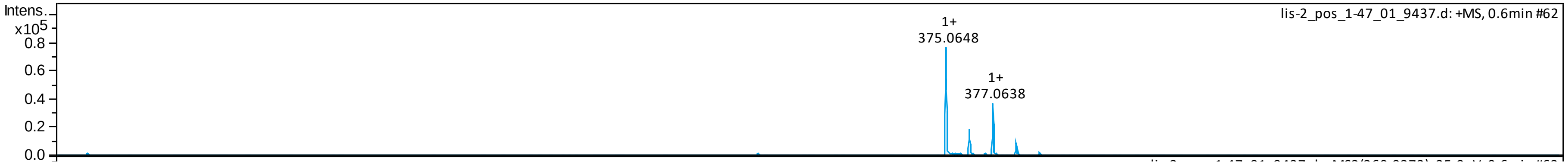

Zoom in 357.0648 emphasis on the isotopic distribution of copper.

Figure S2. ESI-HRMS spectrum of the complex 2.

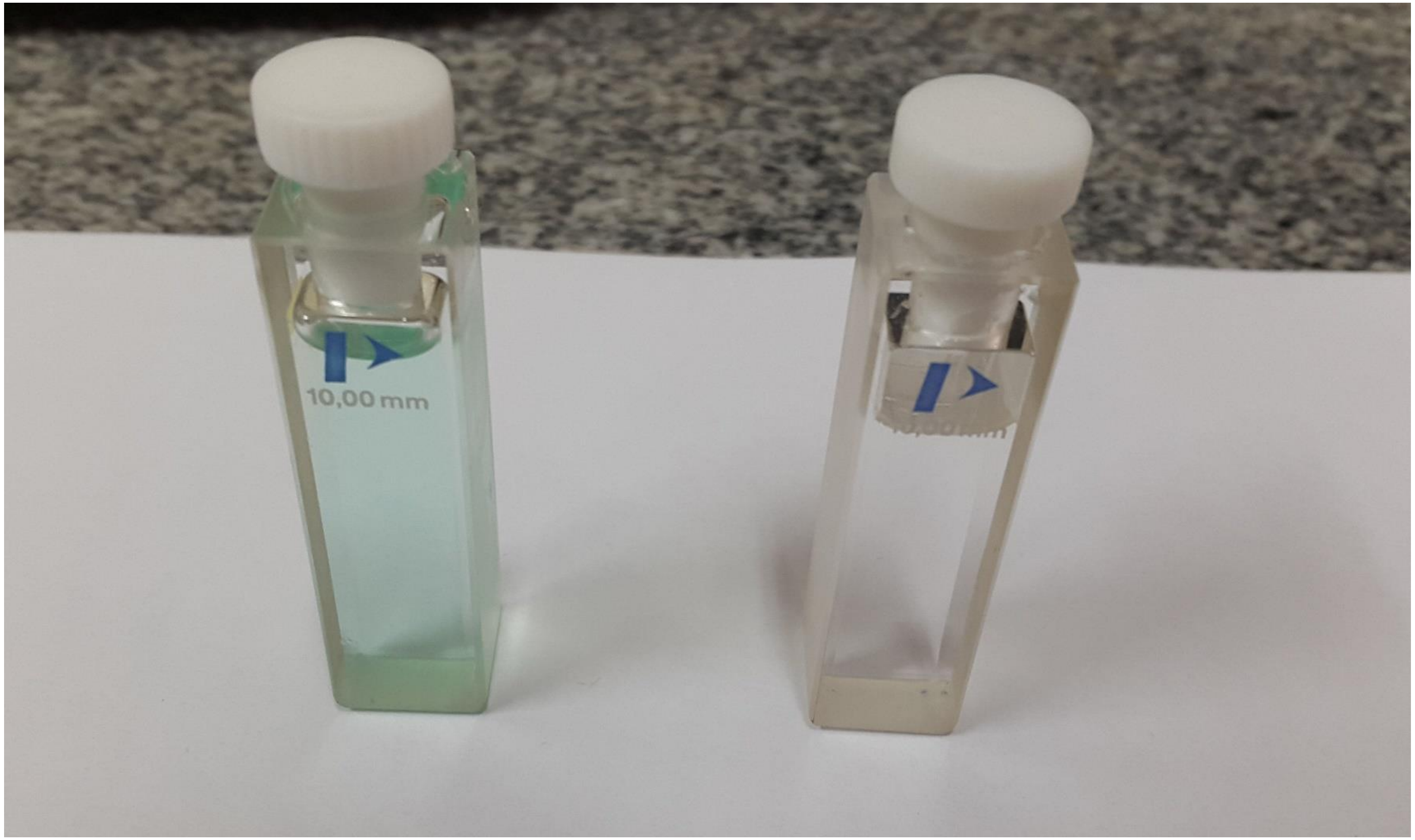

Figure S3. Cuvettes containing the solutions of the complex 1 (light green) and ligand (colorless). Picture taken during the experiment. Complex 2 and respective ligand have been presented similar behavior.

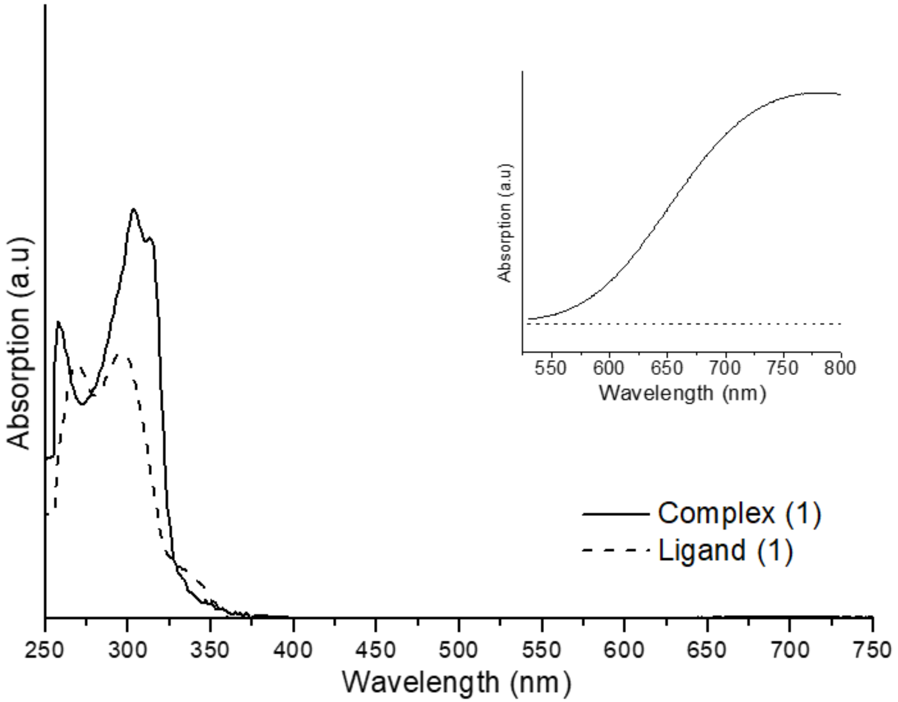

Figure S4. UV-Vis absorption spectra of the complex 1 and respective ligand measured at 298 K. This experiment was performed 36 h after preparing the solutions.

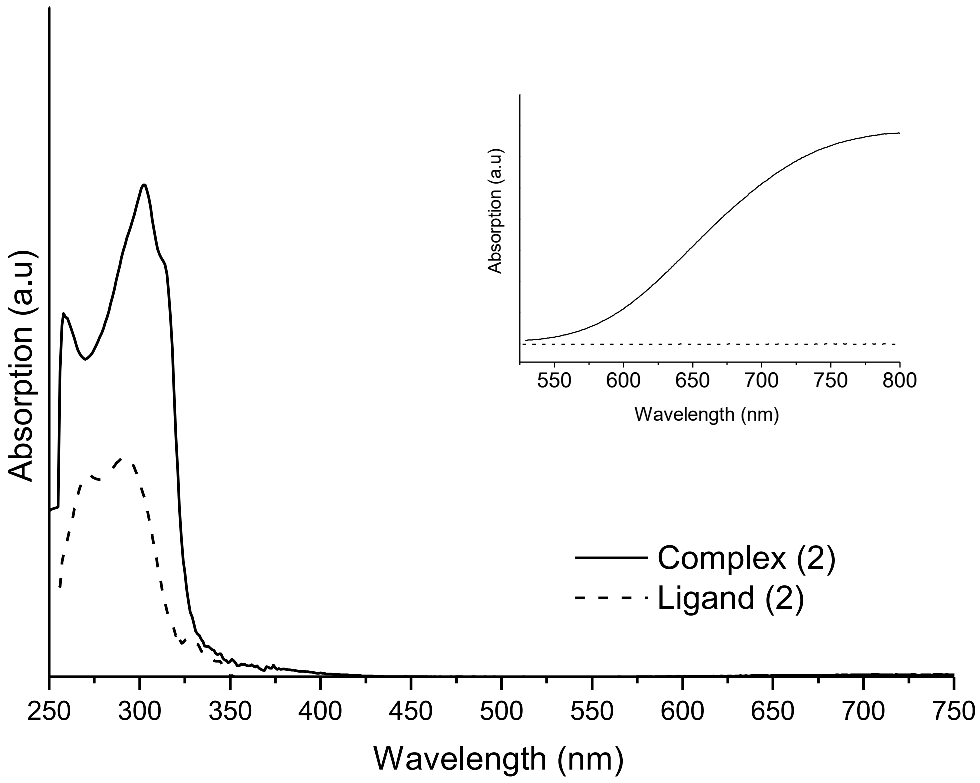

Figure S5. UV-Vis absorption spectra of the complex 2 and respective ligand measured at 298 K. This experiment was performed 36 h after preparing the solutions.

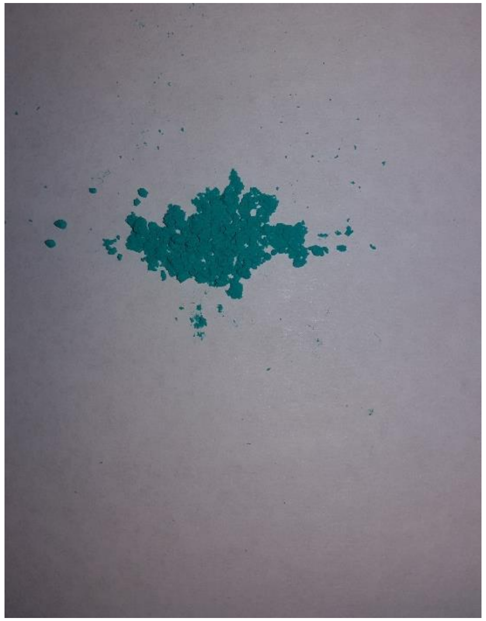

Figure S6. Green solid of the Complex 1 obtained after filtration.

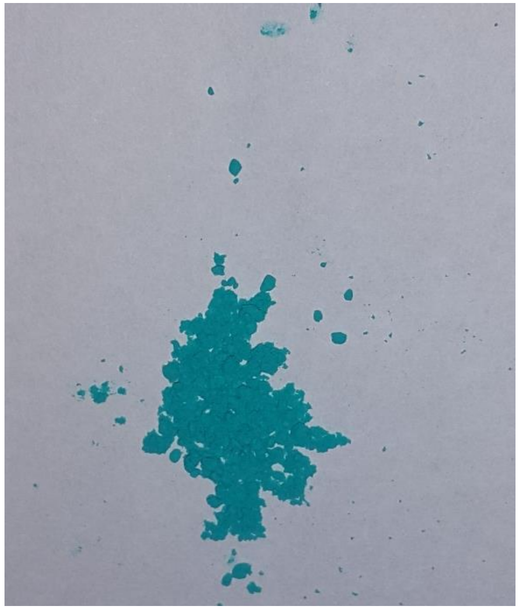

Figure S7. Green solid of the Complex 2 obtained after filtration.

Possible Fragments of the complex 1

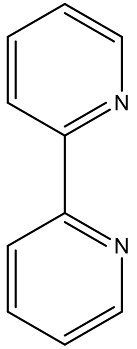

[bipyH$^+$] *m/z* 157.0680

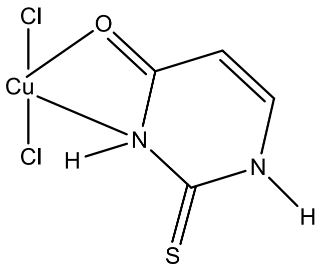

[Cu(L)Cl_2H^+] *m/z* 260.8717

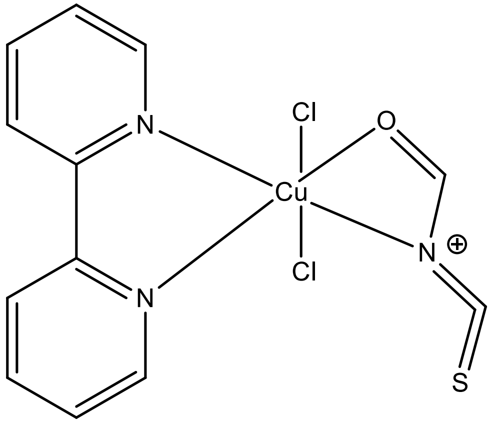

[$C_{12}H_9Cl_2CuN_3OS$]$^+$ *m/z* *375.9134*

Possible fragments of the complex 2

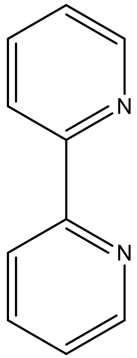

[bipyH$^+$] *m/z* 157.0680

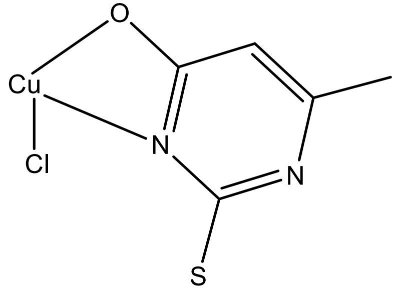

[$C_5H_4ClCuN_2OS$]$^+$ *m/z* 237.9029

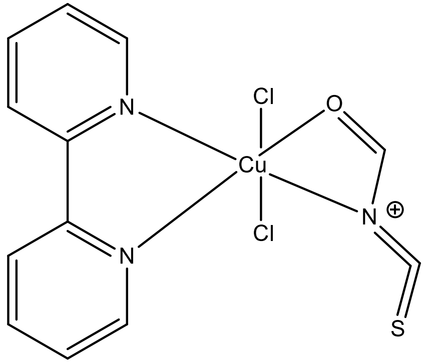

[$C_{12}H_9Cl_2CuN_3OS$] *m/z* *375.9134*
